# A Systematic Review of the Tumor-Infiltrating CD8^+^ T-Cells/PD-L1 Axis in High-Grade Glial Tumors: Toward Personalized Immuno-Oncology

**DOI:** 10.3389/fimmu.2021.734956

**Published:** 2021-09-17

**Authors:** Mahdi Abdoli Shadbad, Zahra Asadzadeh, Negar Hosseinkhani, Afshin Derakhshani, Nazila Alizadeh, Oronzo Brunetti, Nicola Silvestris, Behzad Baradaran

**Affiliations:** ^1^Research Center for Evidence-Based Medicine, Faculty of Medicine, Tabriz University of Medical Sciences, Tabriz, Iran; ^2^Immunology Research Center, Tabriz University of Medical Sciences, Tabriz, Iran; ^3^Student Research Committee, Tabriz University of Medical Sciences, Tabriz, Iran; ^4^Laboratory of Experimental Pharmacology, Istituto Di Ricovero e Cura a Carattere Scientifico (IRCCS) Istituto Tumori Giovanni Paolo II, Bari, Italy; ^5^Medical Oncology Unit, IRCCS Istituto Tumori Giovanni Paolo II, Bari, Italy; ^6^Department of Biomedical Sciences and Human Oncology, University of Bari “Aldo Moro”, Bari, Italy; ^7^Department of Immunology, Faculty of Medicine, Tabriz University of Medical Sciences, Tabriz, Iran

**Keywords:** glioma, tumor-infiltrating lymphocytes, tumor-infiltrating CD8^+^ T-cells, PD-L1, single-cell sequencing, personalized medicine, immune checkpoint, tumor microenvironment

## Abstract

Based on preclinical findings, programmed death-ligand 1 (PD-L1) can substantially attenuate CD8^+^ T-cell-mediated anti-tumoral immune responses. However, clinical studies have reported controversial results regarding the significance of the tumor-infiltrating CD8^+^ T-cells/PD-L1 axis on the clinical picture and the response rate of patients with high-grade glial tumors to anti-cancer therapies. Herein, we conducted a systematic review according to the preferred reporting items for systematic reviews and meta-analyses (PRISMA) statements to clarify the clinical significance of the tumor-infiltrating CD8^+^ T-cells/PD-L1 axis and elucidate the impact of this axis on the response rate of affected patients to anti-cancer therapies. Indeed, a better understanding of the impact of this axis on the response rate of affected patients to anti-cancer therapies can provide valuable insights to address the futile response rate of immune checkpoint inhibitors in patients with high-grade glial tumors. For this purpose, we systematically searched Scopus, Web of Science, Embase, and PubMed to obtain peer-reviewed studies published before 1 January 2021. We have observed that PD-L1 overexpression can be associated with the inferior prognosis of glioblastoma patients who have not been exposed to chemo-radiotherapy. Besides, exposure to anti-cancer therapies, e.g., chemo-radiotherapy, can up-regulate inhibitory immune checkpoint molecules in tumor-infiltrating CD8^+^ T-cells. Therefore, unlike unexposed patients, increased tumor-infiltrating CD8^+^ T-cells in anti-cancer therapy-exposed tumoral tissues can be associated with the inferior prognosis of affected patients. Because various inhibitory immune checkpoints can regulate anti-tumoral immune responses, the single-cell sequencing of the cells residing in the tumor microenvironment can provide valuable insights into the expression patterns of inhibitory immune checkpoints in the tumor micromovement. Thus, administrating immune checkpoint inhibitors based on the data from the single-cell sequencing of these cells can increase patients’ response rates, decrease the risk of immune-related adverse events development, prevent immune-resistance development, and reduce the risk of tumor recurrence.

## Introduction

High-grade glial tumors, e.g., glioblastoma, are among the frequently diagnosed primary brain tumors; however, the prognosis of affected patients with the current treatment is dismal. Although surgery and radio/chemotherapy are considered the first-line therapies for these patients, their response rates have not led to desired outcomes for affected patients. Indeed, a better understanding of the biology of immune cells and the impact of surgery and radio/chemotherapy on the phenotype of immune cells can be essential in increasing their response rates (1).

Traditionally, glial tumors were considered “cold tumors.” The common belief was that immune cells could not cross the blood-brain barrier to develop anti-tumoral immune responses. However, it has been shown that immune cells can cross the blood-brain barrier, and indeed, they have substantial roles in determining the response rates of radiotherapy (2). Recent advances in immuno-oncology have shown that immunotherapy can be a promising approach for treating patients with high-grade glial tumors (3). Nevertheless, the immunosuppressive tumor microenvironment impedes the development of anti-tumoral immune responses. Besides facilitating immune evasion, the immunosuppressive tumor microenvironment can pave the way for tumor proliferation and migration (4).

Inhibitory immune checkpoint axes are among the well-studied culprits in transforming the pro-inflammatory tumor microenvironment to the immunosuppressive one. The PD-L1/programmed cell death protein 1 (PD-1) axis is a well-known inhibitory immune checkpoint axis that can be established between immune cells and tumoral cells. The expression of PD-1 and PD-L1 in the tumor microenvironment have been associated with tumor development (5). Furthermore, growing evidence indicates remarkable associations between PD-1 and other inhibitory immune checkpoints, e.g., V-domain immunoglobulin suppressor of T cell activation (VISTA) and T cell immunoreceptor with Ig and ITIM domains (TIGIT), which can further attenuate anti-tumoral immune responses (6–9).

Accumulating evidence indicates that anti-cancer therapy can substantially alter the tumor microenvironment. Recent findings have demonstrated that chemo-radiotherapy can up-regulate inhibitory immune checkpoints expression and induce a state of exhaustion in tumor infiltration immune cells, e.g., tumor-infiltrating CD8^+^ T-cells (10). Besides, the administration of immune checkpoint inhibitors, e.g., anti-PD-1, has been associated with an increased response rate of anti-cancer therapies in patients with high-grade glial tumors (11). However, the response rates among the affected patients considerably vary. Indeed, some clinical trials have failed to report meaningful benefits of immune checkpoint inhibitors administration in patients with glioblastoma (11, 12). Moreover, despite the well-established anti-tumoral function of CD8^+^ T-cells in eliminating tumoral cells in preclinical studies, there have been controversial results regarding the significance of tumor-infiltrating CD8^+^ T-cells in patients with high-grade glial tumors (13–16). In light of these controversial results, there is a need to clarify the significance of the tumor-infiltrating CD8^+^ T-cells/PD-L1 axis in patients with high-grade glial tumors.

The current study aims to systematically review and sort out the current evidence on the cross-talk between tumor-infiltrating CD8^+^ T-cells with PD-L1 and their impacts on the prognosis, the clinicopathological features, and the response rate of patients with high-grade glial tumors to anti-cancer therapies. Based on the current clinical and preclinical evidence, we also propose a novel strategy for immune checkpoint inhibitor administration, based on single-cell sequencing and personalized medicine principles, to increase the response rates and ameliorate the prognosis of affected patients.

## Methods

The present study was performed according to the PRISMA statements (17). Concerning the PICO, the studied population is patients with high-grade glial tumors. The intervention/exposure is the level of tumor-infiltrating CD8^+^ T-cells/tumoral PD-L1 expression with regard to the prognosis/clinicopathological feature of affected patients. The comparator is the patients with the low level of tumor-infiltrating CD8^+^ T-cells/high PD-L1 expression. The outcome is a better understanding of the impact of the tumor-infiltrating CD8^+^ T-cells/PD-L1 axis on the response rate to anti-cancer therapies and the clinical picture of affected patients.

### Search Strategy

Without restricting to any languages or time, the Web of Science, Scopus, PubMed, and Embase databases were systematically searched to obtain the peer-reviewed records published before 1 January 2021. For this purpose, all fields of records were systematically searched with the following keywords: (“glioma” OR “glioblastoma multiforme” OR “glioblastoma” OR “astrocytoma” OR “ependymoma” OR “subependymoma” OR “oligodendroglioma” OR “oligoastrocytoma” OR “sub-ependymoma” OR “sub ependymoma”) and (“programmed death-ligand 1” OR “PD-L1” OR “PD L1” OR “PDL1” OR “B7-H1” OR “B7 H1” OR “B7H1” OR “CD274” OR “cluster of differentiation 274” OR “CD 274” OR “cluster of differentiation274” OR “B7 homolog 1” OR “PDCD1 Ligand 1” OR “PDCD1LG1” OR “PDCD1L1” OR “HPD-L1”) and (“CD8” OR “CD 8” OR “Cluster of differentiation 8” OR “Cluster of differentiation-8” OR “Cluster of differentiation8” OR “CD8A” OR “T-lymphocyte differentiation antigen T8/Leu-2” OR “CD8 antigen” OR “CD 8 antigen” OR “Leu2 T-Lymphocyte antigen” OR “CD8a molecule” OR “T-cell antigen Leu2” OR “cytotoxic T cell” OR “cytotoxic T lymphocyte” OR “CTL” OR “T-killer cell” OR “cytolytic T cell” OR “CD8+ T-cell” OR “killer T cell”).

### Study Selection

Following the systematic search, the obtained records were reviewed in two phases. In phase I, two authors (NH and ZA) independently screened the relevant papers based on their titles and abstracts. In phase II, the same authors independently reviewed the full text of the remaining papers, along with their supplementary data. Any disagreements were resolved *via* consulting with B.B and consensus.

### Eligibility Criteria

Records with the following eligibility criteria were included in this study : (1) clinical studies, (2) investigations with the objective of assessing the tumor-infiltrating CD8^+^ T-cells and the protein expression of PD-L1 in patients with high-grade glial tumors, (3) studies, which investigated and published the quantified relationship between PD-L1 and tumor-infiltrating CD8^+^ T-cells or the clinicopathological significance of PD-L1 and tumor-infiltrating CD8^+^ T-cells or the prognostic values of PD-L1 and tumor-infiltrating CD8^+^ T-cells in patients with high-grade glial tumors, and (4) studies, which were published in English.

Based on the following criteria, records were excluded from this study: (1) studies that investigated the tumor-infiltrating CD8^+^ T-cells and the protein expression of PD-L1 in patients with low-grade glial tumors, (2) studies that investigated the tumor-infiltrating CD8^+^ T-cells and the expression of tumoral PD-L1 in glial tumors without considering tumor grades, (3) studies that investigated the cross-talk between PD-L1 and circulating CD8^+^ T-cells in the blood of the affected patients, (4) studies that investigated the CD8^+^ T-cells and PD-L1 in a co-culture system, and (5) studies that were based on the data from databases, like The Cancer Genome Atlas (TCGA).

### Data Extraction

The following data were extracted from the included studies: (1) the first author, (2) publication year, (3) the used antibody, (4) the endpoint, (5) the country, (6) the type of high-grade glial tumor, (7) the sample size, (8) the treatment of affected patients, (9) the prognostic values of protein expression of PD-L1/tumor-infiltrating CD8^+^ T-cells, (10) the association between protein expression of PD-L1/tumor-infiltrating CD8^+^ T-cells, (11) the clinicopathological significance of protein expression of PD-L1/tumor-infiltrating CD8^+^ T-cells, and (12) and the cut-off for PD-L1 and tumor-infiltrating CD8^+^ T-cells.

### Quality Assessment of the Included Studies

We applied the criteria of Hayden et al. statements for assessing the quality of the prognostic studies (18). We also utilized the Joanna Briggs Institute (JBI) checklist for assessing the studies that investigated the relationship between PD-L1 and tumor-infiltrating CD8^+^ T-cells in patients with high-grade glial tumors (19).

## Results

### Selected Studies

**Figure 1:**
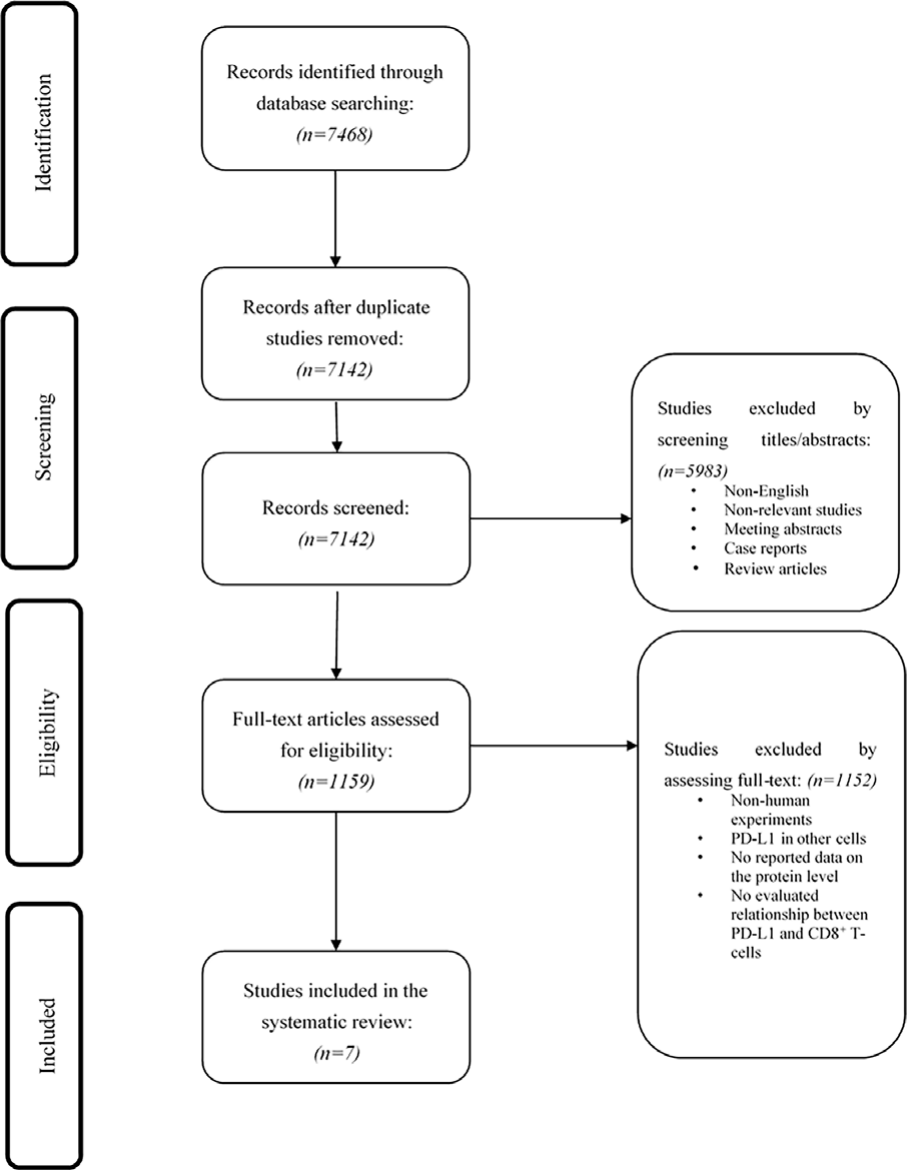
The flow chart of the study selection process.

Our systematic search on PubMed, Scopus, Embase, and Web of Science retrieved 7468 records. After removing duplication records, 7142 records remained. Based on the independent review of two authors in phase I, 5983 studies were excluded. In phase II, two authors independently reviewed the full text of 1159 studies, along with their supplementary data. Finally, based on the full-text assessment of studies, seven papers were included in the qualitative synthesis. **Figure 1** demonstrates the flowchart of literature identification, inclusion, and exclusion.

### Study Characteristics

The included studies were published between 2015 to 2020. Six out of seven studies have investigated high-grade glioma patients (15, 16, 20–23), and one study has investigated high-grade ependymoma patients (24). **Table 1**. demonstrates the general characteristics of the included studies.

**Table 1:** The general characteristics of the included studies.

First author, year	Country	Sample	Glial tumor	Endpoint (s)	Cancer treatment record	CD8+ T-cells cut-off	PD-L1 cut-off	PD-L1 antibody for staining
Su, 2020 (16)	China	47	Glioblastoma	OS of tumor-infiltrating CD8^+^ T-cells and PD-L1 and the association between PD-L1 and tumor-infiltrating CD8^+^ T-cells	Surgery	More than 10 CD8+ T-cells in high-power field	Based on intensity and reactivity	EPR1161 (2)
Nambirajan, 2019 (24)	India	52	High-grade ependymoma	The association between PD-L1 and tumor-infiltrating CD8^+^ T-cells	Radio/chemotherapy	More than 6 CD8^+^ T-cells/mm^2^ in high power filed	1%	SP263
Jan, 2018 (21)	Taiwan	47	Glioblastoma	The OS and PFS of tumor-infiltrating CD8^+^ T-cells and PD-L1	Resection, chemo-radiotherapy, with/without autologous dendritic cell/tumor antigen vaccine	Not clearly mentioned	5%	EPR1161
Plant, 2018 (22)	USA	27	High-grade glioma	The OS of PD-L1 and tumor-infiltrating CD8^+^ T-cells	Not appropriately provided.	Not clearly mentioned	Based on positivity intensity	29E.2A3
Zhang, 2017 (23)	China	17	Glioblastoma	The association between PD-L1 and tumor-infiltrating CD8^+^ T-cells	Not specifically categorized.	Based on pathological scoring	Not clearly defined	28-8
Miyazaki, 2017 (15)	Japan	16	Glioblastoma	The clinical significance, OS, PFS, and survival from the second surgery of PD-L1 and tumor-infiltrating CD8^+^ T-cells	Treated with surgery, radiation, temozolomide, and four patients received a cancer vaccine	Based on pathological scoring	25%	28-8
Berghoff, 2015 (20)	Austria	135	Glioblastoma	The association and clinical significance of tumor-infiltrating CD8^+^ T-cells and PD-L1	Except for the five unknown patients, others were on chemotherapy/investigational agents.	Based on pathological scoring	5%	5H1

OS, Overall survival; PD-L1, Programmed death-ligand 1; PFS, Progression-free survival; and CD, Cluster of differentiation.

We have found a significant increase in the level of tumor-infiltrating CD8^+^ T-cells in the second surgery of glioblastoma patients treated with surgery/fractionated radiotherapy/temozolomide compared to the first resected tumor (P-value=0.009) (**Table 2**). Besides, membranous PD-L1 expression has been more pronounced in the newly diagnosed glioblastoma tissues compared to recurrent glioblastoma tissues (P-value=0.034) (**Table 2**).

**Table 2:** The clinical significance of tumor-infiltrating CD8^+^ T-cells/PD-L1 axis in high-grade glioma patients.

First author, year	Country	Sample size	Clinical significance of the axis	P-value	Cancer treatment history
**Berghoff, 2015 (** 20 **)**	Austria	135	The level of tumor-infiltrating CD8^+^ T-cells is not statistically significantly different in newly diagnosed and recurrent tumors.	0.103	Except for the five unknown patients, others were on chemotherapy/investigational agents.
**Berghoff, 2015 (** 20 **)**	Austria	135	There is no statistically significant correlation between being older/younger than 65 years old and the level of tumor-infiltrating CD8^+^ cells.	0.376	Except for the five unknown patients, others were on chemotherapy/investigational agents.
**Miyazaki, 2017 (** 15 **)**	Japan	16	The level of tumor-infiltrating CD8^+^ cells is significantly increased in the second removal.	0.009	Treated with surgery, radiation, temozolomide, and four patients received a cancer vaccine
**Berghoff, 2015 (** 20 **)**	Austria	135	Diffuse/fibrillary PD-L1 is not statistically associated with tumor recurrence.	0.411	Except for the five unknown patients, others were on chemotherapy/investigational agents.
**Berghoff, 2015 (** 20 **)**	Austria	135	There is no statistical correlation between being older/younger than 65 years old and diffuse/fibrillary PD-L1 expression.	0.383	Except for the five unknown patients, others were on chemotherapy/investigational agents.
**Berghoff, 2015 (** 20 **)**	Austria	135	There is no statistical correlation between being older/younger than 65 years old and membranous PD-L1 expression.	0.612	Except for the five unknown patients, others were on chemotherapy/investigational agents.
**Berghoff, 2015 (** 20 **)**	Austria	135	Membranous PD-L1 expression is more pronounced in initial glioblastoma tumors than recurrent ones.	0.034	Except for the five unknown patients, others were on chemotherapy/investigational agents.
**Miyazaki, 2017 (** 15 **)**	Japan	16	There is no statistically significant difference between membranous PD-L1 in the initial and second resection.	0.187	Treated with surgery, radiation, temozolomide, and four patients received a cancer vaccine

PD-L1, Programmed death-ligand 1; and CD, Cluster of differentiation.

The increased level of tumor-infiltrating CD8^+^ T-cells has been associated with improved overall survival (OS) in high-grade glioma patients who have not been exposed to anti-cancer therapies (P-value<0.05) (**Table 3**). However, increased level of tumor-infiltrating CD8^+^ T-cells has been associated with worse OS from the second surgery glioblastoma patients who have been exposed to anti-cancer therapies (P-value=0.017) (**Table 3**). Besides, the increased level of tumor-infiltrating CD8^+^ T-cells has been associated with inferior survival from the second surgery in glioblastoma patients who have been exposed to anti-cancer therapies (P-value=0.005) (**Table 3**). We have observed that PD-L1 overexpression is associated with inferior OS in glioblastoma patients who have not been exposed to anti-cancer therapies (P-value=0.0119) (**Table 3**).

**Table 3:** The prognostic value of tumor-infiltrating CD8^+^ T-cells/PD-L1 axis in high-grade glioma patients.

First author, year	Country	Sample size	Prognostic value	P-value	HR and 95%CI	Endpoint	Cancer treatment history
**Plant, 2018 (** 22 **)**	USA	27	The level of tumor-infiltrating CD8^+^ T-cells is not statistically associated with OS.	P=0.8	1.0190, and 0.8743-1.1637	OS	Not provided.
**Jan, 2018 (** 21 **)**	Taiwan	20	The level of tumor-infiltrating CD8^+^ T-cells is not statistically associated with OS.	P=0.5	0.725, and 0.284–1.853	OS	Resection and chemo-radiotherapy
**Jan, 2018 (** 21 **)**	Taiwan	20	The level of tumor-infiltrating CD8^+^ T-cells is not statistically associated with PFS.	P=0.73	1.082, and 0.688–1.703	PFS	Resection and chemo-radiotherapy
**Jan, 2018 (** 21 **)**	Taiwan	27	The level of tumor-infiltrating CD8^+^ T-cells is not statistically associated with OS.	P=0.31	1.55, and 0.667–3.601	OS	Resection, chemo-radiotherapy, and autologous dendritic cell/tumor antigen vaccine
**Jan, 2018 (** 21 **)**	Taiwan	27	The level of tumor-infiltrating CD8^+^ T-cells is not statistically associated with PFS.	P=0.36	1.489, and 0.640–3.461	PFS	Resection, chemo-radiotherapy, and autologous dendritic cell/tumor antigen vaccine
**Su, 2020 (** 16 **)**	China	47	The higher level of tumor-infiltrating CD8^+^ T-cells is associated with improved OS.	P<0.05	Not provided	OS	Surgery
**Miyazak, 2017 (** 15 **)**	Japan	16	The higher level of Tumor-infiltrating CD8^+^ T-cells is not associated with PFS.	P=0.495	Not provided	PFS	Treated with surgery, radiation, temozolomide, and four patients received a cancer vaccine
**Miyazak, 2017 (** 15 **)**	Japan	16	The higher level of tumor-infiltrating CD8^+^ T-cells is associated with inferior OS.	P=0.017	Not provided	OS	Treated with surgery, radiation, temozolomide, and four patients received a cancer vaccine
**Miyazak, 2017 (** 15 **)**	Japan	16	The higher level of tumor-infiltrating CD8^+^ T-cells is associated with inferior survival from the second surgery.	P=0.005	Not provided	Survival from the second surgery	Treated with surgery, radiation, temozolomide, and four patients received a cancer vaccine
**Berghoff, 2015 (** 20 **)**	Austria	135	The membranous PD-L1 is not statistically associated with OS.	P=0.724	Not provided	OS	Except for the five unknown patients, others were on chemotherapy/investigational agents.
**Berghoff, 2015 (** 20 **)**	Austria	135	The diffuse/fibrillary PD-L1 is not statistically associated with OS.	P=0.921	Not provided	OS	Except for the five unknown patients, others were on chemotherapy/investigational agents.
**Plant, 2018 (** 22 **)**	USA	27	The tumoral PD-L1 is not statistically associated with OS.	P=0.6	1.0080, and 0.9789 - 1.0371	OS	Not appropriately provided.
**Jan, 2018 (** 21 **)**	Taiwan	20	The fibrillary/membranous PD-L1 is not statistically associated with OS.	P=0.38	0.654, and 0.254 - 1.685	OS	Resection and chemoradiotherapy
**Jan, 2018 (** 21 **)**	Taiwan	20	The fibrillary/membranous PD-L1 is not statistically associated with PFS.	P=0.5	1.435, and 0.498 - 4.137	PFS	Resection and chemoradiotherapy
**Jan, 2018 (** 21 **)**	Taiwan	27	The fibrillary/membranous PD-L1 is not statistically associated with OS.	P=0.1	0.354, and 0.103 - 1.219	OS	Resection, chemo-radiotherapy, and autologous dendritic cell/tumor antigen vaccine
**Jan, 2018 (** 21 **)**	Taiwan	27	The fibrillary/membranous PD-L1 is not statistically associated with PFS.	P=0.248	0.528, and 0.178 - 1.563	PFS	Resection, chemo-radiotherapy, and autologous dendritic cell/tumor antigen vaccine
**Su, 2019 (** 16 **)**	China	47	The PD-L1 overexpression is associated with inferior OS.	P=0.0119	Not provided	OS	Surgery
**Miyazak, 2017 (** 15 **)**	Japan	16	The tumoral PD-L1 is not statistically associated with PFS.	P=0.095	Not provided	PFS	Treated with surgery, radiation, temozolomide, and four patients received a cancer vaccine
**Miyazak, 2017 (** 15 **)**	Japan	16	The tumoral PD-L1 is not statistically associated with OS.	P=0.356	Not provided	OS	Treated with surgery, radiation, temozolomide, and four patients received a cancer vaccine
**Miyazak, 2017 (** 15 **)**	Japan	16	The tumoral PD-L1 is not statistically associated with survival from the second surgery.	P=0.418	Not provided	Survival from the second surgery	Treated with surgery, radiation, temozolomide, and four patients received a cancer vaccine

OS, Overall survival; PD-L1, Programmed death-ligand 1; PFS, Progression-free survival; HR, Hazard ratio; CI, Confidence interval; and CD, Cluster of differentiation.

We have found that PD-L1 expression is inversely correlated with tumor-infiltrating CD8^+^ T-cells in glioblastoma patients who have not been exposed to anti-cancer therapies (r = -0.5064, and P-value= 0.0003) (**Table 4**). Also, we have observed that there is a remarkable relationship between the tumor-infiltrating CD8^+^ PD-1^+^ T-cells with tumor-infiltrating PD-1^+^ lymphocytes in glioblastoma patients (P<0.001) (**Table 4**). Besides, increased PD-1^+^/tumor-infiltrating CD8^+^ T-cell ratio is associated with worse OS and progression-free survival (PFS) in glioblastoma patients (P-value<0.001, and P-value=0.01, respectively) (**Table 4**). The survival of patients with low PD‐L1 expression and low CD8^+^ infiltration is similar to those with high PD‐L1 expression and low CD8^+^ infiltration in glioblastoma patients who have not been exposed to anti-cancer therapies (P-value<0.05) (**Table 4**). Moreover, the low expression of PD-L1 and high level of tumor-infiltrating CD8^+^ T-cells are associated with improved OS in glioblastoma patients who have not been exposed to anti-cancer therapies (P-value<0.05) (**Table 4**). Furthermore, a high PD-1^+^/tumor-infiltrating CD8^+^ T-cells ratio is negatively associated with improved PFS and OS of glioblastoma patients (r =-0.444, P-value<0.02, and r=-0.655, P-value<0.001, respectively) (**Table 4**). We have observed a strong positive association between PD-L1 expression and tumor-infiltrating CD8^+^ T-cell in patients with high-grade ependymomas (P-value=0.03) (**Table 3S**).

**Table 4:** The cross-talk between the PD-L1/PD-1 axis and tumor-infiltrating CD8^+^ T-cells in glioblastoma.

First author, year	Country	Sample size	Tumor type	Cross-talk between the PD-L1/PD-1 axis and tumor-infiltrating CD8^+^ T-cells	P-value	HR, and 95% CI	OR, CI 95%	r
**Berghoff, 2015 (** 20 **)**	Austria	135	Glioblastoma	Tumor-infiltrating PD-1^+^ lymphocytes are correlated with tumor-infiltrating CD8^+^ PD-1^+^ cells.	P<0.001	Not applicable	Not applicable	Not provided
**Berghoff, 2015 (** 20 **)**	Austria	135	Glioblastoma	There is no statistically significant association between diffuse/fibrillary PD-L1 and tumor-infiltrating CD8^+^ T-cells.	P=0.068	Not applicable	Not applicable	Not provided
**Berghoff, 2015 (** 20 **)**	Austria	135	Glioblastoma	There is no statistically significant association between membranous PD-L1 and tumor-infiltrating CD8^+^ T-cells.	P=0.380	Not applicable	Not applicable	Not provided
**Zhang, 2017 (** 23 **)**	China	17	Glioblastoma	There is no statistically significant association between tumoral PD-L1 and tumor-infiltrating CD8^+^ T-cells.	P=0.4959	Not applicable	3, and 0.1270 - 70.8770	Not provided
**Su, 2019 (** 16 **)**	China	47	Glioblastoma	The PD-L1 expression is inversely correlated with tumor-infiltrating CD8^+^ T-cells.	P=0.0003	Not applicable	Not applicable	r = -0.5064
**Su, 2019 (** 16 **)**	China	47	Glioblastoma	The low expression of PD-L1 and high level of tumor-infiltrating CD8^+^ T-cells are associated with improved OS.	P<0.05	Not provided	Not applicable	Not applicable
**Su, 2019 (** 16 **)**	China	47	Glioblastoma	The survival rate of patients with low PD‐L1 expression and low CD8^+^ infiltration is similar to those with high PD‐L1 expression and low CD8^+^ infiltration.	P<0.05	Not provided	Not applicable	Not applicable
**Jan, 2018 (** 21 **)**	Taiwan	20	Glioblastoma	A high PD-1^+^/tumor-infiltrating CD8^+^ T-cell ratio is associated with worse OS.	P<0.001	11.382, and 3.320–35.707	Not applicable	Not applicable
**Jan, 2018 (** 21 **)**	Taiwan	20	Glioblastoma	A high PD-1^+^/tumor-infiltrating CD8^+^ T-cell ratio is associated with worse PFS.	P=0.01	3.458, and 1.304–9.174	Not applicable	Not applicable
**Jan, 2018 (** 21 **)**	Taiwan	27	Glioblastoma	A high PD-1^+^/tumor-infiltrating CD8^+^ T-cell ratio is not statistically associated with OS.	P-value = 0.23	0.567, and 0.224–1.437	Not applicable	Not applicable
**Jan, 2018 (** 21 **)**	Taiwan	27	Glioblastoma	A high PD-1^+^/tumor-infiltrating CD8^+^ T-cell ratio is not statistically associated with PFS.	P=0.44	1.205, and 0.753–1.929	Not applicable	Not applicable
**Jan, 2018 (** 21 **)**	Taiwan	27	Glioblastoma	The PD-1^+^/tumor-infiltrating CD8^+^ T-cell ratio is inversely associated with OS.	P<0.001	Not applicable	Not applicable	r = -0.655
**Jan, 2018 (** 21 **)**	Taiwan	27	Glioblastoma	The PD-1^+^/tumor-infiltrating CD8^+^ T-cell ratio is inversely associated with PFS.	P=0.02	Not applicable	Not applicable	r = -0.444

OS, Overall survival; PD-L1, Programmed death-ligand 1; PFS, Progression-free survival; PD-1, Programmed cell death protein 1; HR, Hazard ratio; CI, Confidence interval; and CD, Cluster of differentiation.

### Evaluating the Bias in the Included Studies

We assessed the included studies concerned with the prognostic values of the PD-L1/tumor-infiltrating CD8^+^ T-cells axis based on the Hayden et al. statement (18). The main risk areas were confounding measurement and outcome measurement (**Table 1S**). Furthermore, we evaluated the remaining studies based on the JBI checklists (19). The main risk areas were addressing potential cofounders (**Table 2S**).

## Discussion

Tumor-infiltrating CD8^+^ T-cells are pivotal cells in eliminating tumoral cells; however, the immunosuppressive tumor microenvironment of solid cancers impedes the development of anti-tumoral immune responses. Indeed, establishing co-inhibitory signals between the tumor-infiltrating immune cells can substantially transform the pro-inflammatory tumor microenvironment into an immunosuppressive one. Besides, the immunosuppressive tumor microenvironment has been implicated in tumor development (5, 25, 26). The PD-L1/PD-1 axis is a well-established inhibitory axis that can attenuate the anti-tumoral immune responses. Besides facilitating immune evasion, tumoral PD-L1 has been implicated in tumor proliferation and migration in glioblastoma. Indeed, PD-L1 knockdown can inhibit tumor growth in mice bearing glioblastoma (27). In the first and second sections, we aim to discuss the clinical significance and prognostic value of this axis and compare our observed results with preclinical and clinical studies. In the third section, we intend to discuss the association between tumor-infiltrating CD8^+^ T-cells with PD-L1 expression in high-grade glial tumors. Finally, we propose a novel strategy to address the shortcomings of immune checkpoint inhibitors that have been reflected in unfavorable objective response rates in multiple clinical trials.

### The Clinical Significance of the Tumor-Infiltrating CD8^+^ T-Cells/PD-L1 Axis

We have found a significant increase in the level of tumor-infiltrating CD8^+^ T-cells in the second resected glioblastoma tumors treated with anti-cancer therapies (paired data). However, there has been no statistically significant change in the level of tumor-infiltrating CD8^+^ T-cells in recurrent glioblastoma patients and newly diagnosed glioblastoma patients treated with chemotherapy/investigational agents (unpaired data) (**Table 2**). Consistent with our results, Yue et al. have reported a strong positive association between increased CD8^+^ T-cells infiltration and positive O^6^-methylguanine DNA methyltransferase (MGMT) expression in glioblastoma patients (28). Indeed, positive MGMT has been associated with chemoresistant tumors in glioblastoma patients (29). These findings are consistent with our observed results regarding the increased infiltration of CD8^+^ T-cells in recurrent glioblastomas. In line with these, preclinical studies have also indicated that anti-cancer therapy of glioma can pave the way for T-cells infiltration. Weichselbaum et al. have shown that radiotherapy can facilitate T-cell infiltration *via* the release of tumor antigens and danger-associated molecular patterns. Indeed, radiotherapy can up-regulate the expression of C-X-C motif ligand 9 (CXCL9) and C-X-C motif ligand 10 (CXCL10), leading to the recruitment of immune cells (30). Moreover, recent findings indicate that radiation can induce major histocompatibility complex (MHC)-I expression, associated with the infiltration of CD8^+^ T-cells into the microenvironment (31).

We have found a remarkable upregulation in PD-L1 in the newly diagnosed glioblastomas compared to the recurrent glioblastomas treated with chemotherapy/investigational agents (**Table 2**). Consistent with this, Heynckes et al. have indicated that temozolomide can inhibit PD-L1 expression in recurrent glioblastoma (32). Besides, it has been reported that PD-L1 expression in recurrent glioblastoma is substantially downregulated following treatment with temozolomide in affected patients (33). Therefore, the insignificant result of the study by Miyazaki et al. might be stemmed from their low sample size. Collectively, based on the current evidence, the level of tumor-infiltrating CD8^+^ T-cells and the expression level of PD-L1 are substantially increased and decreased in the recurrent glioblastomas compared to newly diagnosed glioblastomas.

### The Prognostic Value of Tumor-Infiltrating CD8^+^ T-Cells/PD-L1 Axis

We have found that PD-L1 overexpression can be associated with inferior OS in glioblastoma patients who have not been exposed to anti-cancer therapies (**Table 3**). Consistent with our detected results, Xue et al. have pooled the data from the patients who underwent chemo/radiotherapy after resection with patients treated with other therapeutic modalities and have indicated that PD-L1 can be associated with worse OS in patients with gliomas (34). Nduom et al. have shown that PD-L1 overexpression can be associated with shorter survival in glioblastoma patients (35). Han et al. have indicated that the overexpression of PD-L1 in resected glioblastoma tissues is remarkably associated with the inferior survival of affected patients (36). Besides, Lee et al. have indicated that PD-L1 expression in resected unexposed glioblastoma tissues is associated with worse OS in glioblastoma patients (37).

Regarding the prognostic value of tumor-infiltrating CD8^+^ T-cells, the level of tumor-infiltrating CD8^+^ T-cells might be associated with improved OS in glioblastoma patients who have not been exposed to anti-cancer therapies before. However, tumor-infiltrating CD8^+^ T-cells might be associated with inferior OS and worse survival from the second surgery in glioblastoma patients who were previously exposed to anti-cancer therapies (**Table 3**). Consistent with our detected results, Madkouri et al. have indicated increased infiltration of CD8^+^ T-cells is associated with improved OS of glioblastoma patients who have not been exposed to anti-cancer therapies before (13). Moreover, Kim et al. have demonstrated that increased infiltration of CD8^+^ T-cells in the resected glioblastoma tissues, which have not been exposed to anti-cancer therapies before, can improve the survival of glioblastoma patients (14). In other words, these results have indicated that anti-cancer therapies can continuously lead to the exhaustion of tumor infiltrated CD8^+^ T-cells and pave the way for the transformation of the pro-inflammatory tumor microenvironment into the immunosuppressive one.

Our observed results are also consistent with the preclinical findings. Dai et al. have shown that the combination of anti-PD-1 and temozolomide can substantially decrease tumor size and increase the survival of mice bearing gliomas (10). In mice models of glioblastoma, anti-PD-1 has also remarkably increased the anti-tumoral proprieties of temozolomide *via* down-regulating the expression of lymphocyte-activation gene 3 (LAG-3) and PD-1 (11). Grapin et al. have indicated that radiotherapy can lead to the TIGIT upregulation in the tumor-infiltrating CD8^+^ T-cells (38). In line with this, the combination of fractionated radiotherapy and the administration of immune checkpoint inhibitors can lead to the abscopal effect, resulting in tumor rejection (31). Besides, it has been reported that anti-PD-1 with localized radiation can substantially increase the survival of mice bearing gliomas compared to monotherapy with radiation (39). Consistent with these, Li et al. have highlighted a remarkable PD-1 upregulation in CD8^+^ T-cells following radiation therapy (40). Besides, Dovedi et al. have shown that fractionated radiotherapy can up-regulate PD-1 expression in CD8^+^ T-cells, and the administration of immune checkpoint inhibitors can considerably increase the survival of affected mice (41). Moreover, accumulating evidence indicates that radiation can facilitate the recruitment of regulatory T-cells (Tregs) into the tumor microenvironment. Tregs can up-regulate the expression of interleukin (IL)-10 and transforming growth factor-beta (TGF-β) in the tumor microenvironment. Sharabi et al. have shown that the anti-PD-1 or Treg depletion can substantially increase radiation efficacy in eliminating tumoral cells (42). Besides, Tregs can up-regulate cytotoxic T-lymphocyte-associated protein 4 (CTLA-4) expression and further attenuate the anti-tumoral immune responses. Indeed, CTLA-4 upregulation might be one of the reasons for developing resistance in immune-radiotherapy (43). Besides the beneficial effect of immune checkpoint inhibition on the response rate of glioma radiotherapy, radiotherapy can also increase the permeability of the blood-brain barrier for immune checkpoint inhibitors (44, 45). Therefore, the administration of immune checkpoint inhibitors can increase radiotherapy efficacy and vice versa (41, 42). Collectively, the current evidence indicates that exposure to anti-cancer therapies, e.g., chemo-radiotherapy, can up-regulate inhibitory immune checkpoint molecules in tumor-infiltrating CD8^+^ T-cells, and unlike unexposed patients, increased tumor-infiltrating CD8^+^ T-cells in anti-cancer therapy-exposed tumoral tissues can be associated with the inferior prognosis of affected patients.

### The Cross-Talk Between the PD-L1/PD-1 Axis and Tumor-Infiltrating CD8^+^ T-Cells in High-Grade Glial Tumors

We have observed a significant association between tumor-infiltrating PD-1^+^ lymphocytes with tumor-infiltrating CD8^+^ PD-1^+^ T-cells in glioblastoma patients treated with chemotherapy/investigational agents. Su et al. have shown a significant inverse relationship between PD-L1 expression and the intensity of tumor-infiltrating CD8^+^ T-cells in glioblastoma patients who have not been exposed to anti-cancer therapies (**Table 4**). Nambirajan et al. have reported a strong positive association between PD-L1 expression and the level of tumor-infiltrating CD8^+^ T-cells in patients with high-grade ependymoma (**Table 3S**).

We have found that the combination of low PD-L1 expression and high infiltration of CD8^+^ T-cells is associated with improved OS in glioblastoma patients who have not been exposed to anti-cancer therapies. Also, the survival rate of glioblastoma patients with low PD-L1 expression and low level of tumor-infiltrating CD8^+^ T-cells has been similar to the survival rate of glioblastoma patients with high PD-L1 expression and low level of tumor-infiltrating CD8^+^ T-cells, indicating the critical prognostic value of CD8^+^ T-cells and its phenotype in determining the survival of glioblastoma patients who have not been exposed to anti-cancer therapies. Besides, a high PD-1^+^/tumor-infiltrating CD8^+^ T-cell ratio has been associated with substantially inferior PFS and OS in glioblastoma patients (**Table 4**). Collectively, the presence phenotype of tumor-infiltrating CD8^+^ T-cells has an essential role in determining the survival of glioblastoma patients.

### Single-Cell Sequencing: A Novel Strategy to Address the Daunting Challenges?

Although PD-1 expression in the tumor-infiltrating CD8^+^ T-cells can substantially attenuate anti-tumoral effects of cancer therapies, the tumor microenvironment of glioblastoma is more complicated than its direction can be determined by the expression level of a single inhibitory immune checkpoint molecule. Indeed, various axes, which the PD-L1/PD-1 axis is one of them, determine the fate of anti-tumoral immune responses. Besides the remarkable association between CTLA-4 and PD-1, recent findings have indicated remarkable associations between other inhibitory immune checkpoints, e.g., TIGIT, in gliomas (46). Thus, immunotherapies for glioblastoma patients should be focused on disrupting these inhibitory checkpoints to restore anti-tumoral immune responses.

Furthermore, the low response rate of glioblastoma patients to immune-checkpoint inhibitors compared to melanoma patients also indicates that the glioblastoma tumor microenvironment might not be regulated by a single inhibitory molecule rather a network of the inhibitory immune checkpoints. Nayak et al. have reported that the objective response rate of glioblastoma patients to monotherapy with pembrolizumab is 0% (47). Reardon et al. have shown that the objective response rate of glioblastoma patients to monotherapy with nivolumab is approximately 7.8% (12). Blumenthal et al. have found that monotherapy with pembrolizumab does not bring clinical benefits for patients with brain tumors (48). In contrast to these dismal results, a meta-analysis by Li et al. has shown that PD-1 inhibitors can remarkably improve the OS of melanoma patients (49).

Moreover, the current method of immune checkpoint inhibitors administration can increase the risk of immune-related adverse events development. Administrating immune checkpoint inhibitors without considering the expression patterns of immune checkpoint molecules in the tumor microenvironment cannot effectively stimulate anti-tumoral immune responses in the tumor microenvironment rather can increase the risk of autoimmunity development in healthy tissues (50, 51). Simonelli et al. have reported a glioblastoma patient that nivolumab administration led to severe liver damage (52). Comito et al. have reported a glioblastoma patient that developed aplastic anemia following treatment with nivolumab (53). Therefore, immune checkpoint inhibitors should be administrated according to the immune checkpoints expression patterns in the cells residing in the tumor microenvironment to minimize the risk of immune-related adverse events development (6–8).

**Figure 2:**
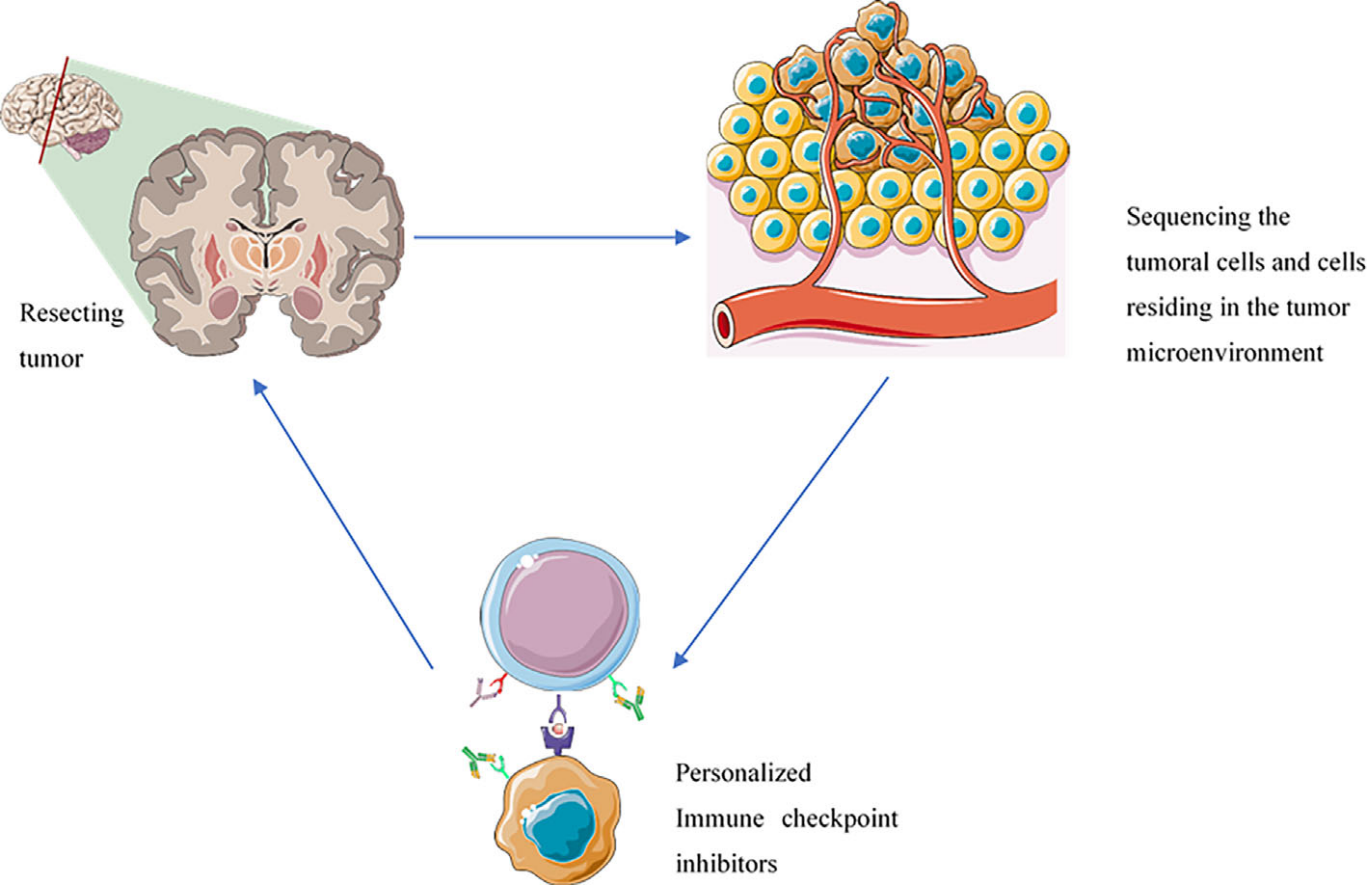
The administration of immune checkpoint inhibitors based on the inhibitory immune checkpoint expression profile of each patient can improve patients’ response rates, decrease the risk of immune-related adverse events development, prevent the immune-resistance development, and reduce the risk of tumor recurrence. The components of this figure were obtained from https://smart.servier.com/.

Single-cell sequencing technology has allowed us to study the cells at the single-cell level. The single-cell sequencing of immune cells, e.g., tumor-infiltrating CD8^+^ T-cells, enables us to demonstrate the expression patterns of various inhibitory immune checkpoint molecules (54). Indeed, the expression profile of the cells in the tumor microenvironment can allow us to design a precise regimen for each patient to increase the response rate of immune checkpoint inhibitors and decrease the risk of immune-related adverse events development following the administration of immune checkpoint inhibitors (54, 55) (**Figure 2**).

Besides, single-cell sequencing can provide valuable insights for predicting the response rate of affected patients to immune checkpoint inhibitors. Although there are established biomarkers for predicting the response rate of patients with solid cancers to immune checkpoint inhibitors, e.g., *BRCA1/2* alteration and mismatch-repair status, the data from single-cell sequencing can study the cells and their origins at the single-cell level and provide with more valuable prognostic biomarkers (56, 57). In this regard, it has been identified that the expression of TCF7 in CD8^+^ T-cells can be a prognostic factor for predicting the response rate of melanoma patients to anti-PD-1 therapy (58).

Zhai et al. have applied single-cell sequencing techniques to investigate the expression of immune checkpoints in resected high-grade glioma. They have found that the expression of the inhibitory immune checkpoints and their pertained ligands in tumoral cells is substantially increased during tumorigenesis (59). Huang et al. have used bioinformatic single-cell sequencing data of immune cells and have demonstrated that CTLA-4 is remarkably up-regulated in natural killer cells (60). Besides the potentiality of this technology in profiling the expression of known inhibitory immune checkpoint molecules and their expression intensities, this technology can also provide new insights about novel inhibitory immune checkpoints in high-grade glial tumors. Li et al. have reported that the sialic acid-binding Ig-like lectin family is a novel inhibitory immune checkpoint in glioma. They have shown that sialic acid-binding Ig-like lectin-16 is functionally similar to PD-L1 and sialic acid-binding Ig-like lectin-5/-7/-9 are functionally similar to immunoglobulin mucin-3 (TIM-3). Besides, their expression levels have been associated with advanced tumor grades in patients with glioma (61). Tan et al. have used bioinformatic single-cell sequencing data of immune cells and have indicated that the expression of sialic acid-binding Ig like lectin 1 is positively correlated with PD-1 and CTLA-4 in glioma (62). A recent clinical trial has used cytometry by time-of-flight technique to identify the reason for the low response rate of glioblastoma patients to pembrolizumab. They have found that the overexpression of CD68 in the tumor microenvironment can be the culprit for the low response rate of glioblastoma patients to pembrolizumab (63). Indeed, the application of single-cell sequencing can provide ample opportunities to investigate the factors that are implicated in immune resistance. Besides, radio/chemotherapy can augment the inhibitory immune checkpoint axes in glioblastoma patients; thus, integrating the results of single-cell sequencing to proscribe immune checkpoint inhibitors can also effectively and precisely improve the response rates of glioblastoma patients.

Despite the promising future of this approach for patients with high-grade glial tumors, this approach has some limitations. The most noticeable limitation of this approach is its complex nature that requires the implantation of high technologies. Its second limitation might be stemmed from the fact that the expression level obtained from sequencing RNAs does not always correlate with their protein expressions because the post-transcriptional modifications can substantially alter the protein expressions. Nevertheless, the recent advances in antibody sequencing and RNA expression and protein sequencing (REAP-seq) technologies can overcome this issue and provide a better insight into the phenotype of cells (55). Its third limitation is that the tumor-microenvironment is highly dynamic, and serial sequencing might be required for optimal results. Thus, follow-up sequencing might be needed. Nevertheless, the promising preclinical results and the recent advances in deep learning might justify its translation and open a new era in the neuro-oncology field (64).

Collectively, PD-L1 overexpression can be associated with the poor prognosis of glioblastoma patients who have not been exposed to anti-cancer therapies. Since anti-cancer therapies, like chemo-radiotherapy, can increase the expression of inhibitory immune checkpoint molecules in tumor-infiltrating CD8^+^ T-cells, exposed glioblastoma tissues to anti-cancer therapies can exhaust tumor-infiltrating CD8^+^ T-cells and these exhausted tumor-infiltrating CD8^+^ T-cells are associated with the inferior prognosis of glioblastoma patients. Nevertheless, the increased infiltration of tumor-infiltrating CD8^+^ T-cells is associated with the improved prognosis of glioblastoma patients who have not been exposed to anti-cancer therapies. Therefore, profiling the expression pattern of inhibitory immune checkpoints in the tumor microenvironment *via* single-cell sequencing technologies and administrating related immune checkpoint inhibitors based on these data can pave the way to increase the response rate of anti-cancer therapies, enhance the efficacy of immune checkpoint inhibitors, decrease the risk of tumor recurrence, improve the immune-resistance state, and reduce the risk of autoimmunity development in the affected patients.

The current systematic review has some strengths: First, all fields of the major electronic databases, i.e., Web of Science, Scopus, PubMed, and Embase, have been searched to minimize the risk of not including eligible studies. Second, the current systematic review has shed light on the controversial results accumulating during the past decade and, *via* a systematic and unbiased approach, have elucidated the significance of the tumor-infiltrating CD8^+^ T-cells/PD-L1 axis in the affected patients. Third, the current systemic review has sorted out the inconsistencies between preclinical and clinical studies and presented novel insights into the tumor microenvironment. Fourth, along with the recent phase II “window-of-opportunity” clinical trial, we have highlighted the potential role of single-cell sequencing in increasing the response rates of anti-cancer therapies, decreasing the risk of immune-related adverse events development, preventing the immune-resistance development, and reducing the risk of tumor recurrence in affected patients. Nevertheless, the current systematic review has some limitations as well. First, the number of included studies has been low because high-grade glial tumors are not as prevalent as other cancers, like breast and lung cancers. Second, our included studies have been limited to the investigations that have been published in English. Third, ideally, studies would apply novel single-cell sequencing-based approaches, e.g., mass cytometry, to study the expression profile of well-established inhibitory immune checkpoints on immune cells; however, so far, the main detection method has been immunohistochemistry (IHC). Therefore, more investigations on the promising potentiality of single-cell sequencing on the profiling of inhibitory immune checkpoints expression are recommended to ameliorate affected patients’ prognosis.

## Conclusion

PD-L1 overexpression can be associated with inferior prognosis in glioblastoma patients unexposed to anti-cancer therapies, e.g., chemo-radiotherapy. We have found that the level of tumor-infiltrating CD8^+^ T-cells can be associated with improved prognosis in glioblastoma patients who have not been exposed to chemo-radiotherapy. Nevertheless, their infiltration level is associated with inferior prognosis in glioblastoma patients who underwent radio/chemotherapy because radio/chemotherapy can up-regulate the expression of inhibitory immune checkpoint molecules, e.g., PD-1, in tumor-infiltrating CD8^+^ T-cells and induce a state of exhaustion in immune cells. Indeed, the expression of inhibitory immune checkpoint molecules in tumor-infiltrating CD8^+^ T-cells decreases the response rate of anti-cancer therapies; thus, the administration of immune checkpoint inhibitors can improve the response rates of radio/chemotherapy approaches. Single-cell sequencing of the cells that reside in the tumor microenvironment can allow us to identify the profile of expressed inhibitory immune checkpoint molecules, which can be used to prescribe related immune checkpoint inhibitors. In this approach, the response rate of affected patients can be improved, and the risk of immune-related adverse events development following administration of immune checkpoint inhibitors can be decreased. However, the technical challenges and the cost of the suggested approach requires further studies to evaluate its cost-effectiveness before its translation into the clinics.

## Author Contributions

MA has come up with the topic, extracted data, and interpreted the data. ZA, NH, AD, NA, and OB have developed the syntax, ran the search, selected the studies, and assessed the quality of included studies. NS and BB have supervised and helped to develop the research question. All authors contributed to the article and approved the submitted version.

## Conflict of Interest

The authors declare that the research was conducted in the absence of any commercial or financial relationships that could be construed as a potential conflict of interest.

## Publisher’s Note

All claims expressed in this article are solely those of the authors and do not necessarily represent those of their affiliated organizations, or those of the publisher, the editors and the reviewers. Any product that may be evaluated in this article, or claim that may be made by its manufacturer, is not guaranteed or endorsed by the publisher.
