# Supplementary material for: A Systematic Review of the Tumor-Infiltrating CD8+ T-Cells/PD-L1 Axis in High-Grade Glial Tumors: Toward Personalized Immuno-Oncology
Source: Front Immunol. 2021 Sep 17;12:734956. doi: 10.3389/fimmu.2021.734956 (PMC8486082; doi:10.3389/fimmu.2021.734956)
Supplement: Supplementary file 1 [file Table_1.docx]

**Table 1S**. Assessing the potential risk of bias of included prognostic studies according to the Hayden et al. guidelines

| First author and year | Study participation | Study attrition | Prognostic factor measurement | Outcome measurement | Confounding measurement and account | Analysis |
| --- | --- | --- | --- | --- | --- | --- |
| Su, 2020 | *** | *** | *** | ** | ** | ** |
| Jan, 2018 | *** | *** | *** | *** | *** | *** |
| Plant, 2018 | ** | *** | *** | *** | *** | *** |
| Miyazaki, 2017 | *** | *** | *** | ** | ** | *** |
| Berghoff, 2015 | *** | *** | *** | ** | ** | *** |

*** Yes, ** Partly, and * No

**Table 2S.** Assessing the potential risk of bias of included prognostic studies according to the JBI checklist

| Items | Zhang, 2017 | Nambirajan, 2019 |
| --- | --- | --- |
| 1. Were the groups comparable other than the presence of the studied factor? | Yes | Yes |
| 2. Were cases and controls matched appropriately? | Yes | Yes |
| 3. Were the same criteria used for the identification of cases and controls? | Yes | Yes |
| 4. Was exposure measured in a standard, valid and reliable way? | Yes | Yes |
| 5. Was exposure measured in the same way for cases and controls? | Yes | Yes |
| 6. Were confounding factors identified? | Unclear | Unclear |
| 7. Were strategies to deal with confounding factors stated? | Unclear | Unclear |
| 8. Were outcomes assessed in a standard, valid and reliable way for cases and controls? | Yes | Yes |
| 9. Was the exposure period of interest long enough to be meaningful? | Yes | Yes |
| 10. Was appropriate statistical analysis used? | Yes | Yes |

**Table 3S**. The cross-talk between the PD-L1/PD-1 axis and tumor-infiltrating CD8^+^ T cells in high-grade ependymoma.

| First author, year | Country | Sample size | The cross-talk/clinicopathological significance of CD8^+^ tumor-infiltrating lymphocyte and tumoral PD-L1 | P-value | OR, and 95%CI |
| --- | --- | --- | --- | --- | --- |
| Aruna Nambirajan, 2019 | India | 52 | There is a strong positive between tumoral PD-L1 and CD8^+^ tumor-infiltrating lymphocytes. | P= 0.0373 | 5.8824, and 1.1101 - 31.1708 |
